# Declaration on mental health in Africa: moving to implementation

**DOI:** 10.3402/gha.v7.24589

**Published:** 2014-06-16

**Authors:** Abdallah S. Daar, Marian Jacobs, Stig Wall, Johann Groenewald, Julian Eaton, Vikram Patel, Palmira dos Santos, Ashraf Kagee, Anik Gevers, Charlene Sunkel, Gail Andrews, Ingrid Daniels, David Ndetei

**Affiliations:** 1 Dalla Lana School of Public Health and Department of Surgery, University of Toronto and Grand Challenges Canada, Toronto, Canada; 2 Faculty of Health Sciences, University of Cape Town, Cape Town, South Africa; 3 Department of Public Health and Clinical Medicine, Umeå University, Umeå, Sweden; 4 Strategic Initiatives, Stellenbosch Institute for Advanced Study, Stellenbosch, South Africa; 5 CBM International, West Africa Regional Office, Lomé, Togo; 6 Centre for Global Mental Health, London School of Hygiene and Tropical Medicine, London, UK; 7 Sangath Centre, Provorim, Goa, India; 8 Mental Health Department, Center for Applied Psychology and Psychometric Tests, Ministry of Health, Mozambique; 9 Department of Psychology, Stellenbosch University, Stellenbosch, South Africa; 10 Gender & Health Research Unit, South African Medical Research Council, Pretoria, South Africa; 11 Department of Psychiatry & Mental Health, University of Cape Town, Cape Town, South Africa; 12 Awareness, Advocacy & Communications Officer, Central Gauteng Mental Health Society, Johannesburg, South Africa; 13 Office of the Director General, National Department of Health, Pretoria, South Africa; 14 Cape Mental Health, Observatory, South Africa; 15 Department of Psychiatry, University of Nairobi, Nairobi, Kenya; 16 Africa Mental Health Foundation, Montreal, Canada

**Keywords:** Africa, mental health, WHO action plan, roadmap, basic services, policy, implementation, stigma, human rights, post-2015 Agenda, UN General Assembly

## Abstract

Urgent action is needed to address mental health issues globally. In Africa, where mental health disorders account for a huge burden of disease and disability, and where in general less than 1% of the already small health budgets are spent on these disorders, the need for action is acute and urgent. Members of the World Health Organization, including African countries, have adopted a Comprehensive Mental Health Action Plan. Africa now has an historic opportunity to improve the mental health and wellbeing of its citizens, beginning with provision of basic mental health services and development of national mental health strategic plans (roadmaps). There is need to integrate mental health into primary health care and address stigma and violations of human rights. We advocate for inclusion of mental health into the post-2015 Sustainable Development Goals, and for the convening of a special UN General Assembly High Level Meeting on Mental Health within three years.

## On behalf of

**Table:** List of Signatories

Firstname lastname	Organization
Atalay Alem	College of Health Sciences, Addis Ababa University, Ethiopia
Astrid Berg	Department of Child Psychiatry, University of Cape Town, South Africa
Yomi Esan	Department of Psychiatry, University of Ibadan, Nigeria
Hendrik Geyer	Director, Stellenbosch Institute for Advanced Study (STIAS), South Africa
Oye Gureje	Department of Psychiatry, UCH, University of Ibadan, Nigeria
Karen Hofman	Director, Priority Cost Effective Lessons for Systems Strengthening, University of Witwatersrand, School of Public Health, South Africa
Lars Jacobsson	Department of Psychiatry, Umeå University, Sweden
Lotta Jernström	Department of Health and Social Care, SKL, Sweden
Sylvia Kaaya	Department of Psychiatry, Muhimbili University, Tanzania
Lou-Marie Kruger	Department of Psychology, Stellenbosch University, South Africa
Bernard Lategan	Former Director, STIAS
Ingrid le Roux	Philani Child Health & Nutrition Project, Cape Town, South Africa
Nasser Loza	Behman Psychiatric Hospital, Cairo, Egypt
Crick Lund	Centre for Public Mental Health, University of Cape Town, South Africa
Sandra Marais	Researcher, South African Medical Research Council
Keymanthri Moodley	Department of Psychiatry, Stellenbosch University, South Africa
Bronwyn Myers	South African Medical Research Council
Maud Olofsson	Former Deputy Prime Minister of Sweden
Inge Petersen	PRIME South Africa & Department of Psychology, University of Kwazulu-Natal, South Africa
Soraya Seedat	Department of Psychiatry, Stellenbosch University, South Africa
Dan Stein	Department of Psychiatry & Mental Health. University of Cape Town, South Africa
Leslie Swartz	Department of Psychology, Stellenbosch University, South Africa
Stephen Tollman	Agincourt, School of Public Health, University of Witwatersrand, South Africa
Mark Tomlinson	Department of Psychology, Stellenbosch University, South Africa
Lize Weich	Department of Psychiatry, Stellenbosch University, South Africa

On 24 and 25 February 2014, a group of people with a common interest in mental health met at the Stellenbosch Institute for Advanced Study (STIAS) in South Africa at a roundtable meeting to address the topic: *Mental Health Challenges in Sub-Saharan Africa: Moving to Implementation*.

Participants included representatives of groups of interest such as persons with psychosocial disabilities, NGOs, policymakers, academics, research funders, service providers, and others from East, West, South, and North Africa, as well as colleagues from Sweden, Canada, the US, Germany, and the World Health Organization (WHO).^1^

Together with others who participated in planning and at a previous workshop at STIAS, we recognized that in spite of mental, neurological, and substance use disorders constituting a very high burden of disease globally, and that depression is the leading cause of disability throughout the world, these disorders have not been given sufficient attention. In Africa, where mental disorders also account for a huge proportion of burden of disease, in general less than 1% of the already minimal national health budgets are spent on these disorders. In communities in which persons with psychosocial disabilities live, and even in the health care system, the affected persons, their families, and caregivers are frequently stigmatized and experience social exclusion and discrimination; and it is often assumed that little can be done to address their circumstances. However, a growing body of scientific evidence shows that much can be done for treatment, at moderate additional costs, and with significant economic benefits to countries, while at the same time reducing suffering and improving, and often saving, the lives of those who are affected.

At a global level, the 194 member states of the WHO (including those from Africa) have adopted the Comprehensive Mental Health Action Plan (MHAP) with the objectives of advancing the mental health agenda in the world. This plan is supported by technical tools like the mhGAP Intervention Guide for non-specialist health settings, to assist in scaling up services. In Africa, these provide important opportunities for country-led intervention.

We believe that action is urgently needed not just by governments and other groups as set out below but also by international donors who contribute to health budgets and influence health policy, the mental health professional community, medical and public health schools, research institutions, and research funding bodies.

Recognizing that there is no health without mental health, that widespread poverty results in greater vulnerability to mental illness, and that mental health therefore deserves particular attention on the African continent, we believe that:

1. Africa has an historic opportunity now to improve the mental health and wellbeing of its citizens.
2. There is an urgent need for political vision, commitment, and leadership at the highest level to encourage national dialogue on mental health. Governments should take the lead, while working with and supporting an inclusive, cross-sectoral, multi-stakeholder approach that has been found to be critical for dealing effectively with mental conditions and in addressing the social circumstances that create disability associated with these.
3. While supportive legislation and access to mental health services are crucial, there is an urgent need to address stigma, social exclusion and discrimination as dealing with these greatly contributes to improving the quality of life. Communities that include service users, their families, and other stakeholder groups must play a major role in bringing about these positive changes.
4. There is an opportunity for every African government to build on the MHAP to develop a National Mental Health Strategy and Plan (Roadmap). We support the evidence and experience that indicate that such a plan must:
  - Encompass the principle of parity in providing resources for mental and physical health alike;
  - Integrate mental health care services at all levels of the health system, with a focus on integration into primary health care;
  - Include provision of resources for training, supervising, and supporting different cadres of health and other personnel with an emphasis on task-sharing;
  - Take a life-course approach, recognizing that there are different needs at different stages in life such as pregnancy, infancy, childhood and older age, and that investment in early intervention can reduce later disability;
  - Allow for targeting of actions to address the specific needs of groups such as women, the very poor, the homeless, and so on, many of whom have been historically neglected. Such specific focus is necessary because these groups may have different risk factors, disease prevalence, and help-seeking behaviors;
  - Be person-centered and holistic, providing psychological and social care as well as improving access to biomedical services;
  - Specifically respond to the mental health needs that arise as a consequence of violence in society, especially against women and children;
  - Include provision of care that is evidence-based and culturally appropriate; and
  - Pay particular attention to the link between mental health and other health and development priorities like HIV/AIDS and Maternal and Child Health. Integrating mental health into other health and development initiatives provides an opportunity to improve outcomes in other sectors (1, 2), while allowing efficient investment in mental health through these other programs.
    We noted the experience of some countries like South Africa and Ethiopia who have progressed in developing national mental health policy frameworks and strategic plans. Together with the WHO Comprehensive Mental Health Action Plan, such examples are a useful template to be modified appropriately by other African countries. These and other relevant plans can be accessed for reference through the WHO MiNDbank resource: http://www.who.int/entity/mental_health/mindbank/en/index.html.
    Furthermore, acknowledging that governments have a moral and legal obligation to safeguard the human rights of all their citizens, including those who suffer from mental conditions, we also recommend that:
5. In parallel with developing a national strategic plan, governments must ensure availability of all essential medications and basic services for mental health care for all their citizens; and
6. It is necessary for governments to have a robust legislative response to counter inhumane practices against those who are suffering, such as institutionalization, imprisonment, isolation, discrimination in access to public goods, and other violations of their human rights.

We also recognize that African governments and civil society organizations have the immediate opportunity to join the leadership of those advocating for the inclusion of mental health in the UN post-2015 Sustainable Development Goals.

Furthermore, we urge African governments, civil society organizations, and others to advocate strongly for the convening of a special UN General Assembly High Level Meeting on Mental Health within 3 years.

In providing an evidence base for such advocacy, we recommend that African governments should foster implementation research that focuses on sustainability and scaling-up of services at affordable cost; support research on mental health integration into primary care; and facilitate gathering of high-quality epidemiological data on mental disorders, including better integration of mental health in routine national health management information systems. In this regard, sharing of knowledge, experience, and best practices among African countries could be very valuable, especially if done on a subregional level.

In conclusion and in pursuit of these interventions, we recommend engagement through Africa-wide mental health networks made up of various stakeholder groups, including communities of persons with psychosocial disabilities, their families, and their caregivers.

## Footnotes

1 We sincerely thank WHO's Dr. Shekhar Saxena for his many valuable contributions.
